# The influence of genetic structure on phenotypic diversity in the Australian mango (*Mangifera indica*) gene pool

**DOI:** 10.1038/s41598-022-24800-7

**Published:** 2022-11-30

**Authors:** Melanie J. Wilkinson, Risa Yamashita, Maddie E. James, Ian S. E. Bally, Natalie L. Dillon, Asjad Ali, Craig M. Hardner, Daniel Ortiz-Barrientos

**Affiliations:** 1 School of Biological Sciences, The University of Queensland, Brisbane, QLD 4072 Australia; 2 Australian Research Council Centre of Excellence for Plant Success in Nature and Agriculture, The University of Queensland, Brisbane, QLD 4072 Australia; 3 Queensland Alliance for Agriculture and Food Innovation, The University of Queensland, Brisbane, QLD 4072 Australia; 4 Queensland Department of Agriculture and Fisheries, Mareeba, QLD 4880 Australia

**Keywords:** Agricultural genetics, Evolutionary biology, Plant breeding, Population genetics, Phylogenetics

## Abstract

Genomic selection is a promising breeding technique for tree crops to accelerate the development of new cultivars. However, factors such as genetic structure can create spurious associations between genotype and phenotype due to the shared history between populations with different trait values. Genetic structure can therefore reduce the accuracy of the genotype to phenotype map, a fundamental requirement of genomic selection models. Here, we employed 272 single nucleotide polymorphisms from 208 *Mangifera indica* accessions to explore whether the genetic structure of the Australian mango gene pool explained variation in trunk circumference, fruit blush colour and intensity. Multiple population genetic analyses indicate the presence of four genetic clusters and show that the most genetically differentiated cluster contains accessions imported from Southeast Asia (mainly those from Thailand). We find that genetic structure was strongly associated with three traits: trunk circumference, fruit blush colour and intensity in *M. indica*. This suggests that the history of these accessions could drive spurious associations between loci and key mango phenotypes in the Australian mango gene pool. Incorporating such genetic structure in associations between genotype and phenotype can improve the accuracy of genomic selection, which can assist the future development of new cultivars.

## Introduction

Horticultural tree crops are vital for sustainable food production^1^ and ornamental and industrial use. Tree crops can be more sustainably cultivated over time than annual field crops, thus helping to manage food supply for an increasing world population^2^. To create new tree fruit cultivars with improved productivity and quality, we must develop breeding technologies that overcome biological limitations to their production. Tropical species, such as mango, are often large and vigorous^3^, leading to canopies that rapidly outgrow their orchard space. This generates shade, providing a breeding ground for disease^4^. To avoid the adverse effects of tree size, trees are traditionally planted at low density and heavily pruned each year^4^, leading to a reduction in overall production per hectare and an increased cost per unit output. Consequently, a quest to breed smaller, less vigorous trees while maintaining high yields of quality fruit is underway^5,6^. Such efforts will produce mango that can be grown in intensive, high-density orchards that produce more fruit per hectare^7^.

Traditional tree breeding is slow, as evaluations require an assessment of phenotypic performance in mature trees over many years to account for the effects of variable spatial and temporal environments on phenotypic diversity. These evaluations, in combination with a long juvenile phase (typically 2–4 years^4^), can result in a selection process of up to or longer than 10 years from field planting^8^, making the rapid development of new cultivars unfeasible. The time for cultivar development could be reduced by predicting future phenotypic performance in young individuals using genomic selection, as demonstrated in apples^9^, sweet cherry^10^ and strawberry^11^. Genomic selection uses genotype to phenotype maps from a training population to predict phenotypic variation in untested populations using marker data^12,13^. Thus, once a genomic selection model has been created, the length and expense of phenotyping key traits may be reduced. Genomic selection for tree size and vigour of progeny could therefore improve the breeding process and reduce the cost of mango breeding compared to traditional breeding approaches.

The primary assumption of genomic selection is that genetic markers are closely linked on a chromosome with the causative loci that contribute to the trait of interest^14^. In general, the closer the marker is to the causative loci, the more accurate the genotype to phenotype map. However, genetic structure can create statistical associations between loci that are not physically linked. This occurs because evolutionary forces such as migration, drift and mutation can make allelic combinations between unlinked loci more common than expected by chance^15^. Genetic structure can therefore create spurious associations between genetic markers and traits. Furthermore, genetic structure is often prevalent in modern crops, particularly those moving across the world via human migrations, which likely experienced drastic fluctuations in population size and suffered from inbreeding after crossing genetically related individuals with favourable traits^16^.

Differentiating uninformative loci due to genetic structure from those linked to causative loci is a common problem observed in genetic studies of human disease^17,18^ and the study of trait evolution across diverse taxa^19,20,21,23^. Fortunately, we can improve the accuracy of the genotype to phenotype map by accounting for genetic covariation between traits and markers due to genetic structure^24–26^, a practice that can potentially improve the quality of horticultural breeding programs that start from highly variable germplasm collections. Here, we evaluate the assumption that horticultural trait variation segregates independently from genetic structure using *Mangifera indica* in the gene pool of the Australian Mango Breeding Program.

Mango is a major horticultural tree crop worldwide, yet an understanding of the domestication history is still debated. The centre of origin of the genus *Mangifera* is Southeast Asia, but the origin of the species *M. indica* is still under question. Based on the fossil record, Mukherjee^27^ and Blume^28^ suggested that mango originated in the Malay Archipelago less than 2.58 million years ago. However, recent molecular taxonomy suggests it evolved within a large area of Northwest Myanmar, Bangladesh and Northeast India^29^. From this area, human migration and trading led to the dispersion of mangoes to many regions of the world^30^.

Several studies have evaluated the genetic structure of domesticated mango^31,32,33,34,35,36,38^. Yet, to our knowledge, there have been no published studies on the effects of genetic structure on phenotypic variation in mango accessions. One study with 60 mango accessions from India accounted for genetic structure in a marker trait analysis^35^, however, Lal et al.^35^ did not assess the effect of genetic structure on their genotype to phenotype map. Without understanding the effect of genetic structure on phenotypic diversity, we do not know whether we are creating false associations between genetic markers and key mango traits. Here, we directly examined the effects of genetic structure on the creation of spurious associations between genetic markers and three traits – trunk circumference (a proxy for tree size), fruit blush colour and intensity – in the Australian mango gene pool. We assessed 272 SNP markers genotyped in 208 *M. indica* accessions imported worldwide and revealed statistical associations between genetic markers and traits arising from genetic structure. These results will help guide future studies incorporating genetic structure into their genomic selection models.

## Results

### Genetic structure in the Australian mango gene pool

**Figure 1:**
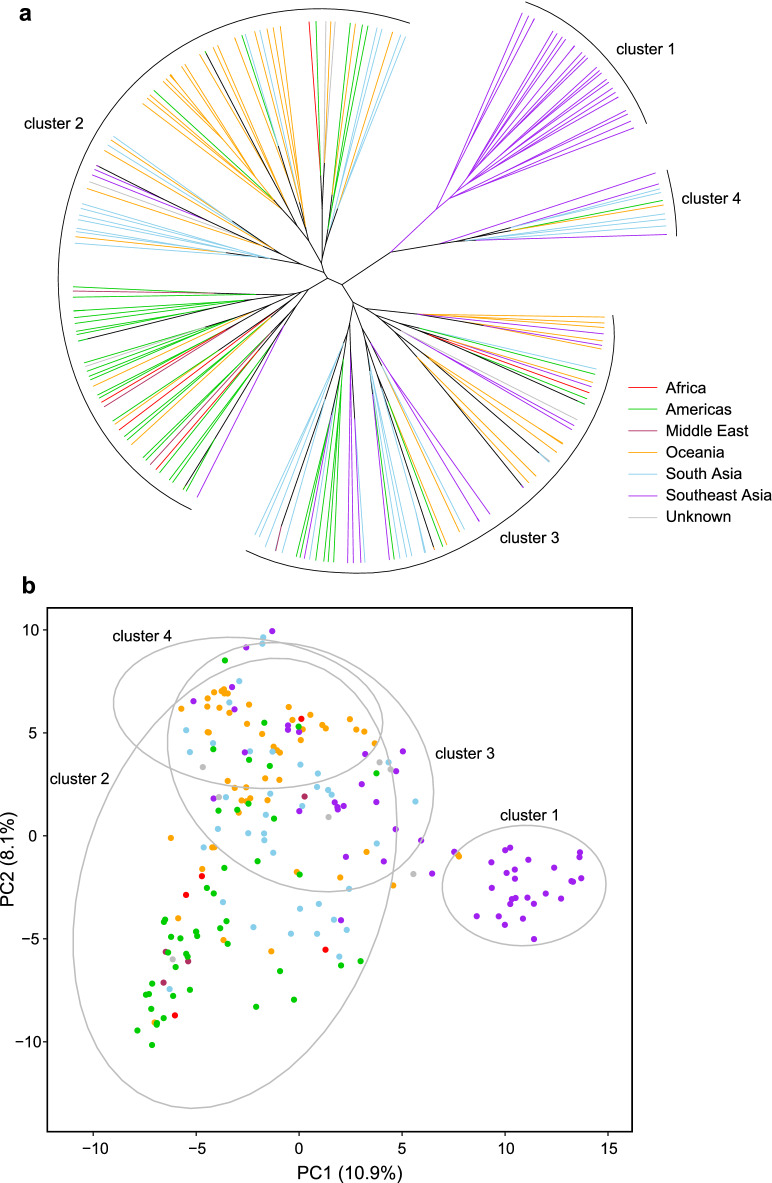
Genetic structure analyses for K = 4 of the 208 accessions of *M. indica* from six geographical regions across the world. (**a**) A circular dendrogram showing the hierarchical cluster analysis using complete linkage clustering. Each branch represents an individual with the colour of the branch representing the geographical region the sample was imported into Australia from. (**b**) Principal components analysis, where the ellipses (95% probability) represent the four clusters from the hierarchical cluster analysis.

**Figure 2:**
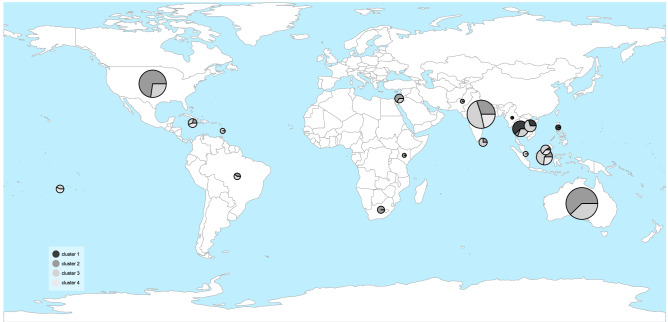
Genetic structure across geography of the 208 *M. indica* accessions. Cluster numbers (K = 4) were determined using a hierarchical cluster analysis (Fig. 1). The size of each pie chart reflects the number of accessions imported from each country. The world map was created in “rworldmap” v1.3–6 R-package (https://cran.r-project.org/web/packages/rworldmap/).

Genetic structure was found in both a hierarchical cluster analysis (HCA) and a principal component analysis (PCA) across all 208 *M. indica* accessions (Fig. 1). Consistent with a recent origin of all accessions, the HCA created a dendrogram with only short branches in the centre (Fig. 1a), indicating few genetic differences separate the clusters. The optimal number of genetic clusters was K = 4, as indicated by the HCA and the elbow plot. The elbow plot from the HCA shows diminishing returns in the amount of variance explained after five clusters (Fig. S1). In the dendrogram, cluster 1 is the most genetically differentiated cluster, which only contains accessions imported from Southeast Asia. Cluster 1 is most distinct from clusters 2 and 3. In contrast, cluster 4 is more similar to cluster 1 (Fig. 1a) and contains a mixture of samples across geographical regions (e.g., South Asia, Southeast Asia, Americas, and Oceania; Table 1; Fig. 2). In the reduced principal component (PC) space (Fig. 1b), genetic clusters largely overlap, with South Asian accessions (mostly Indian accessions) primarily concentrated in the centre of the multivariate space. Genetic clusters from Southeast Asia, the Americas, and Oceania occur towards the edges of the genotypic space, with Southeast Asia distinctly separated in the PC1 axis.

**Table 1:** The number of accessions of *M. indica* from each country of import and their assigned genetic clusters from the hierarchical cluster analysis for K = 4 calculated from 272 biallelic SNPs. Countries have been grouped into six geographical regions of import.

Country	Country code	cluster 1	cluster 2	cluster 3	cluster 4	Country total
**Africa**						
East Africa	EAF			1		1
Kenya	KEN		1			1
South Africa	ZAF		3			3
**Americas**						
Brazil	BRA		1	1		2
Jamaica	JAM		1	1	1	3
Saint Lucia	LCA			1		1
United States of America	USA		32	8		40
**Middle East**						
Israel	ISR		3	1		4
**Oceania**						
Australia	AUS		37	15		52
French Polynesia	PYF			1	1	2
**South Asia**						
India	IND		14	15	5	34
Pakistan	PAK		1			1
Sri Lanka	LKA		1	2		3
**Southeast Asia**						
Indonesia	IDN		2	6	2	10
Malaysia	MYS	1		2	1	4
Malesia	MLS		1	2	1	4
Myanmar	MMR	1				1
Philippines	PHL	3				3
Singapore	SGP			1		1
Thailand	THA	18	1	3		22
Vietnam	VNM	5		4		9
unknown			4	3		7
Cluster total		28	102	67	11	208

**Figure 3:**
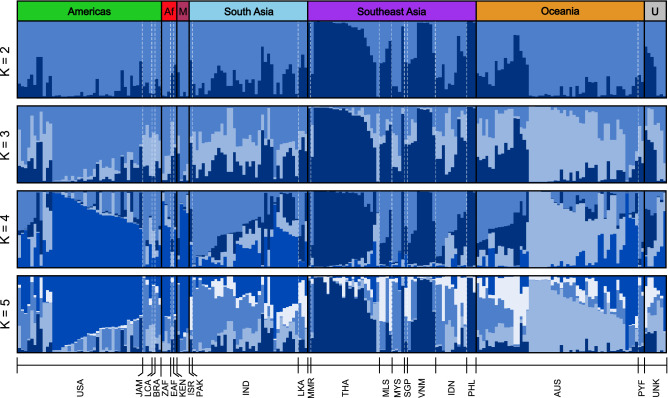
Genetic structure of 208 *M. indica* individuals using STRUCTURE for K = 2 to K = 5. Each bar represents an individual with the shades of blue representing the ancestry proportions to each cluster. Individuals are sorted by geographical region (black lines), where Af = Africa, M = Middle East and U = unknown, and country (white dotted lines). Refer to Table 1 for information on each country code.

In agreement with the HCA and PCA results above, we identified genetic clusters across the 208 *M. indica* accessions (Fig. 3) using the Bayesian clustering approach implemented in STRUCTURE^39^. Most accessions contained large amounts of admixture or shared ancestral polymorphism, where portions of their genome were assigned to different genetic groups. When genetic differentiation was separated into only two groups (K = 2, see Methods), Southeast Asia formed one group, while all other accessions were in a second group (Fig. 3). Relaxing this constraint to K = 3 revealed the Americas and Oceania accessions each form a group. Populations are almost indistinguishable when K is larger than 4. Consistent with the elbow plot discussed above, the Evanno method^40^ and the log probability of K values show that K = 4 was the optimal number of clusters (Fig. S2). Most accessions show signatures of admixture as indicated by diversity from multiple groups. Admixture signals are particularly pronounced in accessions from South Asia, mainly those from India, which do not form a distinct genetic group with any K-value.

Together, the HCA, PCA and STRUCTURE results suggest that mango accessions of the Australian mango gene pool consist of four genetic groups. Southeast Asian accessions are most differentiated relative to the rest of the world, suggesting that these accessions might have evolved differently, thus creating a heterogenous gene pool for cultivar creation in the Australian Mango Breeding Program.

### Patterns of genetic diversity across the Australian mango gene pool

Genetic diversity analyses revealed high levels of heterozygosity and variable patterns of inbreeding across regions (Table 2). Levels of expected heterozygosity (H_E_) and observed heterozygosity (H_O_) were high across the world, with the Americas having the highest levels of observed heterozygosity (H_O_ = 0.49) and Southeast Asia having the lowest (H_O_ = 0.39). Accessions from the Americas contain an excess of heterozygote individuals (i.e., a negative inbreeding coefficient; F_IS_ = − 0.11; 95% CI − 0.13 to − 0.08). On the other hand, accessions from Southeast Asia are mildly inbred (i.e., a positive inbreeding coefficient; F_IS_ = 0.08; 95% CI 0.06 to 0.11). Private alleles were absent in all regions, indicating either a large intermixing population or the presence of ancestral polymorphisms that have not been sorted across geography.

**Table 2:** Genetic diversity for 208 *M. indica* accessions across six geographic regions using 272 SNPs.

Region	H_O_	H_E_	F_IS_ (95% CI's)	Pr
Africa	0.46	0.45	− 0.02 (− 0.08 to 0.05)	0
Americas	0.49	0.44	− 0.11* (− 0.13 to − 0.08)	0
Middle East	0.46	0.45	− 0.01 (− 0.08 to 0.05)	0
Oceania	0.43	0.42	− 0.02 (− 0.05 to 0.00)	0
South Asia	0.45	0.45	0.00 (− 0.03 to 0.02)	0
Southeast Asia	0.39	0.42	0.08* (0.06 to 0.11)	0

H_O_, observed heterozygosity; H_E_, expected heterozygosity; F_IS_, inbreeding co-efficient with 95% confidence intervals, where *CI’s do not overlap with 0; Pr, the number of private alleles.

Genetic differentiation comparisons showed variable patterns of F_ST_ between genetic clusters and between regions of import. Comparisons between regions have low levels of F_ST_, which range from − 0.016 to 0.112 (Table 3a). Southeast Asia and the Middle East, closely followed by the comparison between Southeast Asia and the Americas, showed the highest level of genetic differentiation (F_ST_ = 0.112 and 0.107, respectively). In contrast, F_ST_ between clusters ranged from 0.051 to 0.286, with cluster 1 comparisons having the highest values (Table 3b). Overall, there is low genetic divergence amongst regions of the Australian mango gene pool and high genetic divergence between genetic clusters.

**Table 3:** Pairwise F_ST_ for *M. indica*. a) F_ST_ estimates for the geographical regions and b) clusters. F_ST_ estimates are below the diagonal, 95% confidence interval above the diagonal are based on 1000 bootstrap replicates. Clusters (K = 4) were identified using hierarchical cluster analysis (Fig. 1).

Region	Africa	Americas	Middle East	Oceania	South Asia	Southeast Asia
**a**						
Africa	–	− 0.012 to 0.004	− 0.036 to 0.005	0.022 to 0.055	0.010 to 0.035	0.062 to 0.095
Americas	− 0.004	–	− 0.008 to 0.012	0.051 to 0.070	0.036 to 0.053	0.092 to 0.121
Middle East	− 0.016	0.002	–	0.059 to 0.099	0.009 to 0.036	0.088 to 0.134
Oceania	0.039*	0.060*	0.079*	–	0.031 to 0.046	0.070 to 0.095
South Asia	0.022*	0.044*	0.022*	0.038*	–	0.049 to 0.069
Southeast Asia	0.078*	0.107*	0.112*	0.082*	0.060*	–

Cluster	1	2	3	4		
**b**						
1	–	0.151 to 0.191	0.135 to 0.174	0.250 to 0.322		
2	0.171*	–	0.043 to 0.059	0.127 to 0.170		
3	0.153*	0.051*	–	0.050 to 0.074		
4	0.286*	0.148*	0.062*	–		

*95% confidence intervals do not overlap with 0.

### Genetic structure and region of import influence phenotypic diversity

Phenotypic correlation analyses revealed associations between fruit blush colour and intensity but not between them and trunk circumference. Trunk circumference, a continuous trait, was highly variable at 9 years, ranging from 27 to 70 cm, while categorical fruit traits were less variable (see Fig. S3 for photos of each fruit blush colour and intensity category). In a single-factor linear model, fruit blush colour and intensity were strongly correlated (LR χ^2^ = 373.168, df = 4, *p* < 0.0001, R^2^ = 0.61). However, given that 39% of mango accessions lacked fruit blush colour and therefore lacked fruit blush intensity, we removed ‘no blush’ and retested the association. It led to a significant yet weaker association between the fruit traits (LR χ^2^ = 95.077, df = 3, *p* < 0.0001, R^2^ = 0.28), indicating the importance of no blush in our understanding of the genetics of blush in mango. We found no correlation between trunk circumference and fruit blush colour (Fig. S4; F_4,203_ = 1.093, *p* = 0.3613, R^2^ = 0.02) and trunk circumference and fruit blush intensity (Fig. S5; F_4,203_ = 1.473, *p* = 0.2118, R^2^ = 0.03), suggesting trunk circumference is likely to be genetically independent of these fruit traits.

**Figure 4:**
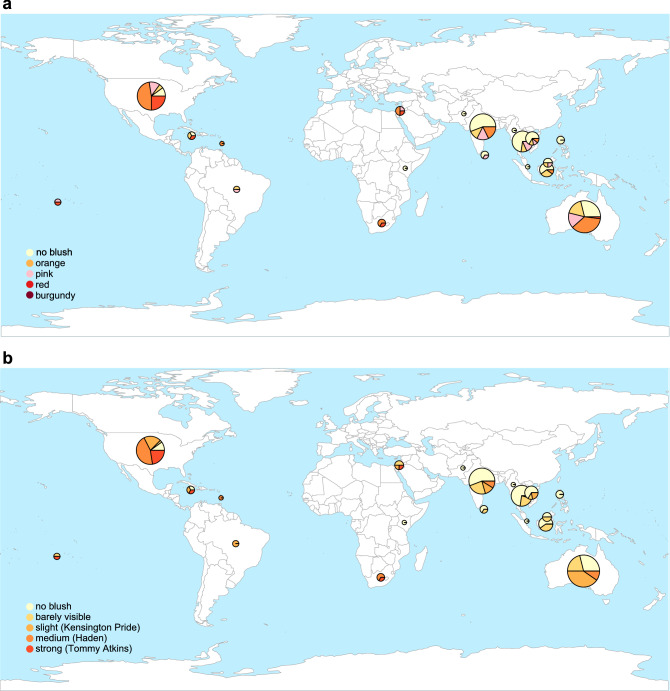
Fruit blush colour and intensity across geography of the 208 *M. indica* accessions. (**a**) Fruit blush colour is split into five categories. (**b**) Fruit blush intensity increases from no blush to strong blush on an ordinal scale, where the accessions in brackets best reflect the colour intensity. The size of each pie chart reflects the number of accessions imported from each country. The world map was created in “rworldmap” v1.3-6 R-package (https://cran.r-project.org/web/packages/rworldmap/).

Fruit blush traits are strongly associated with the region of import in the Australian mango gene pool. In single trait linear models, region of import showed a significant effect on fruit blush colour (Fig. 4a; LR χ^2^ = 77.768, df = 12, *p* < 0.0001, R^2^ = 0.14) and fruit blush intensity (Fig. 4b; LR χ^2^ = 98.936, df = 3, *p* < 0.0001, R^2^ = 0.18), but not trunk circumference (F_3,188_ = 1.970, *p* = 0.1200, R^2^ = 0.03). Of the regions that had more than ten samples, trunk circumference ranged from a mean of 48.1 ± 1.8 (n = 38) in South Asia to a mean of 52.5 ± 1.3 (n = 46) in the Americas (Table S1). For fruit blush colour (Table S2), 67% of accessions from Southeast Asia had no blush colour, while only 11% from the Americas had no blush, with most having red blush (43%). For fruit blush intensity (Table S3), the Americas had 41% of accessions with a medium blush intensity that resembled the Haden accession. In comparison, Oceania had 39% of accessions with slight blush intensity resembling the Kensington Pride accession. Contrastingly, 94% of Southeast Asian accessions and 82% of South Asian accessions had no blush or barely visible blush intensity.

Fruit blush colour, intensity and trunk circumference were all associated with the four clusters assigned in the HCA. Cluster assignment had a significant effect on fruit blush colour (LR χ^2^ = 47.074, df = 12, *p* < 0.0001, R^2^ = 0.08) and the presence of blush (LR χ^2^ = 28.046, df = 3, *p* < 0.0001, R^2^ = 0.10), where 18% of individuals in cluster 1 had blush, whereas 70% and 69% of individuals from clusters 2 and 3 had blush, respectively. Cluster 1 is more likely to have lower blush intensity than the other clusters when the ‘no blush’ category is excluded (LR χ^2^ = 12.274, df = 3, *p* = 0.0065, R^2^ = 0.04; odds ratios between cluster 1 and clusters 2 to 4 ranged from 3.8 to 10.5). Finally, cluster had a significant effect on trunk circumference (F_3,204_ = 18.410, *p* < 0.0001, R^2^ = 0.21), where cluster 1 (mean = 52.3 ± 1.5, n = 28) and cluster 2 (mean = 53.7 ± 0.8, n = 102) had the largest trunk circumference and cluster 4 had the smallest (mean = 36.8 ± 2.9, n = 11). Overall, we expect that genetic diversity and factors specific to the region of import will likely influence the genotype to phenotype map of these key mango traits.

## Discussion

Genetic structure arises from evolutionary processes such as mutation, migration and genetic drift, which drive shifts in allelic frequency that could cause statistical associations between random genetic markers and traits^41^. Such variation arising from genetic structure is often confounded with loci contributing to trait variation in association studies^17,18,19,20,21,23^, which can misrepresent the genotype to phenotype map assumed in genomic selection models. Our study shows how genetic structure in *M. indica* can lead to statistical associations between genetic markers and three phenotypic traits measured in this study – trunk circumference, fruit blush colour and intensity. This suggests that the genetic architecture of these horticultural traits contains noise arising from the conflation of phenotypic and historical differences in the Australian mango gene pool. Such noise can create spurious associations that hinder the selection of new cultivars, so we recommend that future studies in mango breeding take this into consideration.

Genetic variability and divergence in the Australian mango gene pool can be understood in two ways. On the one hand, accessions imported from different regions are weakly differentiated. On the other hand, genetic clusters are strongly differentiated, implying the existence of clear genetic groups. Results described in Fig. 2 reveal that genetic clusters are distributed across regions, implying that their genetic structure is shared across the world. The net effect of this nested relationship between geographic region and genetic cluster is low F_ST_ values amongst the regions yet high levels of F_ST_ amongst genetic clusters. This relationship can be used to hypothesise the causes of genetic divergence in the Australian mango gene pool.

In our study, cluster 1 (containing only Southeast Asian countries) comprises the most genetically differentiated accessions from across the world. Previous studies support this observation; Warschefsky and von Wettberg^31^ showed that accessions from Southeast Asia cluster together in a STRUCTURE plot, and Dillon et al.^32^ reached a similar conclusion using genetic distance analyses of 254 mango accessions. Surprisingly, we did not find private alleles (exclusive alleles) to Southeast Asia, such as those found in Warschefsky and von Wettberg^31^ (74 private alleles from a total of 364 SNPs; 20%). It is unclear what is driving this difference in the number of private alleles between the two studies. However, some of the factors that could be contributing to the variation in sampled loci include different cultivars, contrasting sequencing techniques (SNP chip vs. Restriction site associated DNA markers), different approaches for calling variants, and for filtering of the minor allele frequency^42,43^. The genetic differentiation observed between Southeast Asia and the rest of the world might have been driven by regional cultural differences. For example, in Southeast Asia, mangoes are incorporated into savoury dishes, which might have led to the selection of immature mangoes that stay green while ripening and therefore lack blush^31^. On the other hand, red blush is favoured around the world^44^, likely accentuating genetic differentiation between accessions from Southeast Asia and the rest of the world.

Artificial selection for these cultural preferences may have driven some of the genetic differentiation identified in the Australian mango gene pool. It is well accepted that selecting one trait can incidentally lead to the evolution of other traits through genetic linkage^45,46^. The genetic architecture of selected traits will largely determine the extent of this correlated evolution. In this study, we show that fruit blush colour and intensity are highly correlated, which might imply a shared genetic architecture. Therefore, selection for either of these traits could partially drive the evolution of the other. For example, the evolution of low blush intensity, but not trunk circumference, might have arisen from selection of low levels of blush colour in Southeast Asia. Selection of polygenic traits and recruitment of pleiotropic genes can also affect levels of genetic differentiation across the genome. Selection for trunk circumference, which is a polygenic trait^47^, might therefore drive changes in allelic frequencies across many loci. In contrast, fruit colour pigments and their levels, are often controlled by fewer loci in simpler biochemical pathways^48–50^. In general, we expect genes controlling plant growth and development^51–53^ to be important drivers of genetic differentiation between accessions and merit further attention considering the influence of the genetic architecture of selected traits on population structure.

Polyembryony could have contributed to the origin of genetic differences between Southeast Asia and other accessions. Southeast Asian accessions are typically polyembryonic, where all but one (the zygotic embryo) of the multiple somatic embryos are genetically identical to the maternal parent. Polyembryony is likely to easily be maintained under moderate to strong selection as it is thought to be inherited through a single dominant gene^54,55^. A high level of polyembryony can freeze the genetic diversity in a population, as instead of allowing hybridisation and creating unique individuals through recombination, it propagates genetically identical individuals^56^. Polyembryony can therefore create genetic bottlenecks if only a fraction of the original genetic diversity is propagated, consistent with the signature of inbreeding in Southeast Asian accessions we found in this study. Furthermore, previous studies have found genetic clustering of mango accessions according to their ability to produce polyembryonic seed^57,58^. However, embryo type is conflated with geographic region in these studies, where Southeast Asian accessions dominate the polyembryony types. Therefore, without future work teasing apart the contribution of polyembryony and geographic region than we lack an understanding of the various causes of polyembryonic selection and inbreeding on the genetic diversity of tree crops.

Genetic diversity and partitioning of genetic structure influence prediction accuracy in genomic selection models across horticultural crops^16,24,26,59–61^. For instance, increasing genetic diversity by using a variety of races or genetic clusters in the training and validation sets produced higher prediction accuracies in rice, sorghum^16^ and wheat^61^. But genetic diversity is known to reduce prediction accuracy when estimation error is high, which occurs in small populations or when there is low marker density^61,62^. By definition, using markers close to the causative variants will augment prediction accuracy during breeding; however, this is hard to achieve with low-density genotyping techniques such as SNP chips and Genotyping by Sequencing. With sequencing that covers the entire genome (e.g., whole genome sequencing), factors influenced by linkage disequilibrium can be better controlled, such as finding markers in tight linkage with causative loci. As such, population size, marker density, the genetic structure of the population, and the genetic architecture of the chosen traits will play a significant role in the accuracy of genomic selection models.

To ameliorate the adverse effects of genetic structure in genomic selection models, there are two major approaches used across horticultural crops^16,24,26,59,61^. The first approach includes principal components from genetic structure analyses as covariates in the model^63–66^. However, this method can double-count genetic structure because some elements are included in the model through the genomic relationship matrix^67^. Another common approach for accounting for genetic structure in genomic selection models is ensuring an equal contribution across genetic clusters in training and validation sets. This stratified sampling approach has been shown to increase prediction accuracy in sorghum^16^ and maize, and could be an effective method in the Australian mango gene pool. In general, choosing the most accurate genomic selection model will largely depend on the breeding population's genetic structure and the number of samples.

## Conclusion

The results of this study reveal that a horticultural species spread across the world has a genetic structure that can create statistical associations between three key traits and genetic markers. To remove the effects of spurious markers, breeders should fully characterise the genetic structure of their breeding population. This will allow them to incorporate sample stratification to improve the performance of genomic selection models. Together with best practices of genomic selection (e.g., whole genome sequencing and large population size), these considerations can improve the genotype to phenotype map to assist in choosing individuals with accurate breeding values and help advance future parental selection. We hope our study encourages other horticultural breeding programs to follow similar methods.

## Methods

### Ethics statement

All plant material used in this research was sourced and collected from the Walkamin Research Station, Queensland (17.1341°S, 145.4271°E), where trees are held as a living collection. The Department of Agriculture and Fisheries granted permission as stated in the National Tree Genomics Program – Phenotype Prediction project (AS17000) for use and collection of materials from mango trees from their government station. This study complies with relevant institutional, national, and international guidelines and legislation.

### Accessions

A total of 208 *M. indica* accessions were used from the gene pool collection of the Australian Mango Breeding Program at Walkamin Research Station. These accessions were imported from 21 countries across six geographical regions and were grafted onto the uniform polyembryonic rootstock, Kensington Pride. See Table 1 for the complete list of countries and sample sizes.

### Genotyping

To identify some of the genotypic diversity in the Australian mango gene pool, we used the genotypes from Kuhn et al.^68^. DNA isolation for these genotypes was described in Kuhn et al.^69^. Briefly, young leaf samples were collected from Walkamin Research Station and the glasshouse at Mareeba Research Facility, Queensland (17.0075°S, 145.4295°E). DNA was extracted using 20 mg of fresh sample with the Qiagen Plant DNeasy kit. SNP genotyping was performed on these DNA samples using the Fluidigm EP-1 platform with 384 biallelic SNP markers. Finally, 272 SNP markers were selected for further analyses, where 236 markers belong to one of 20 linkage groups (7–20 markers per linkage group), and the location of the remaining 36 markers in the genome is unknown^68^. Genotypically identical individuals across the 272 SNPs were consolidated, leaving 208 mango accessions for the analyses. On average, 98% of the 272 SNPs used in this study were successfully genotyped in every accession.

### Hierarchical cluster analysis

To examine the genotypic clustering of the mango accessions due to genotypic similarity, we performed a hierarchical cluster analysis (HCA) of the 208 *M. indica* accessions. First, pairwise genetic distances between all accessions were calculated using the percentage method by the “ape” v5.3 R-package^70^. The HCA was conducted by “stats” v3.6.2 R-package with complete linkage clustering. This computes all pairwise dissimilarities between the accessions in a cluster and accessions in another cluster and considers the largest value of these dissimilarities as a distance between the two clusters. To assess the optimal number of clusters, we used the elbow method^71^, which plots the total within-cluster sum of squares (WSS) against the number of clusters to show the ‘elbow’ where the WSS rate of decrease slows and indicates diminishing returns with more clusters^72^.

### Principal components analysis

We assessed the major patterns of genetic similarity among the 208 mango accessions in multivariate space using a principal components analysis (PCA) with 272 SNPs. Missing SNP data were imputed using the regularised iterative PCA algorithm with the “missMDA” v1.17 R-package^73^. The PCA was performed using the “stats” v3.6.2 R-package^74^. Ellipses were constructed for each of the four clusters in the HCA to identify the position of every individual in a cluster in multivariate space with 95% probability.

### Structure analysis

We determined levels of admixture between all 208 *M. indica* accessions with STRUCTURE v2.3.4^39^. STRUCTURE is a Bayesian Markov chain Monte Carlo (MCMC) program that assigns individuals into genetic clusters (K) based on their genotypes by assuming Hardy Weinberg equilibrium within a cluster. It gives each accession an admixture coefficient to depict the proportion of the genome originating from a particular K cluster. We ran the admixture model and the correlated allele frequency model^75^ with ten independent runs of 100,000 burn-in and 100,000 MCMC iterations for K = 1 to K = 7. We visually inspected summary statistics of MCMC runs to ensure convergence of model parameters. Results were summarised and plotted in the “pophelper” v2.2.7 R-package^76^. The optimal K value (which represents the most likely number of sub-populations) was estimated by the Evanno method^40^, which uses the second-order rate of change in the log probability of data between successive K values in the R-package StructureSelector^77^. The optimal K value was also estimated using LnP(K), the mean log probability of the data. We also followed suggestions by Pritchard et al.^78^ and Lawson, et al.^79^ and plotted the lowest K values that capture the primary structure in the data.

### Genetic diversity and genetic differentiation

To examine the level of differentiation between the clusters and geographical regions, Weir and Cockerham’s pairwise F_ST_ and 95% confidence intervals were estimated by “hierfstat” v0.4.22 R-package^80^. Each accession was assigned to a cluster based on the HCA, and each country of import was grouped into six geographic regions. We calculated 95% confidence intervals for each pairwise comparison using 1000 bootstrap replicates. Significance was determined by whether the confidence interval overlapped with 0.

Measures of genetic diversity were calculated for all 208 *M. indica* accessions for each of the six geographic regions. A genind object was created in “adegenet” v2.1.2 R-package^81,82^ for input into “hierfstat” v0.4.22 R-package^80^ to calculate observed heterozygosity (H_o_), expected heterozygosity (H_E_) and the inbreeding coefficient (F_IS_). To determine whether F_IS_ was significantly different from 0, we calculated 95% confidence intervals for each pairwise comparison using 1,000 bootstrap replicates. The number of private alleles (Pr) was calculated with the “poppr” v2.8.6 R-package^83,84^.

### Phenotyping

To capture some of the phenotypic diversity in the Australian mango gene pool, we measured three traits in all 208 mango accessions – trunk circumference, fruit blush colour and fruit blush intensity. Trunk circumference was used as a proxy for tree size, as it has been found to be a strong indicator of tree size in other tree crops^85–87^. Trunk circumference was measured 10 cm above the graft when the trees were 9 years old at Walkamin Research Station. After maturity (> 5 years old), fruit blush colour and intensity were assessed once a year using ten ripe fruits from each mango accession for at least 2 years. Fruits were taken from the outside of the tree, where they are exposed to full sun and have well developed blush. Fruit blush included five categories: no blush, orange, pink, red and burgundy (Fig. S3a). Fruit blush intensity was recorded as five ordinal variables increasing in colour intensity (Fig. S3b), where the accessions in brackets best reflect the colour intensity: no blush, barely visible, slight (Kensington Pride), medium (Haden) and strong (Tommy Atkins).

### The effect of region of import and genetic structure on phenotypic diversity

Tests of association were undertaken to examine the relationship between traits. Chi-square likelihood ratios were used to test phenotypic association amongst the categorical traits of fruit blush and intensity. We then performed the same analysis with the ‘no blush’ category removed to test whether the association remains. A linear model was performed to test for an association between trunk circumference and fruit blush colour, and also trunk circumference and fruit blush intensity.

To understand the effect of region of import on both genotype and phenotype in the Australian mango gene pool, we tested its association with genetic structure and phenotypic diversity. We investigated the influence of geographic region on phenotypic diversity for three key mango phenotypes – trunk circumference, fruit blush colour and intensity. We performed a likelihood-ratio chi-square test for fruit blush colour (categorical) and intensity (ordinal) against the region of import and a linear model for trunk circumference. Region of import was the explanatory variable in each model and included the regions shown in Table 1, excluding unknown regions (n = 7) and regions with low samples sizes, including the Middle East (n = 4), and Africa (n = 5).

We then tested for an effect of genetic structure on the three phenotypes using the optimal cluster assignment of K = 4 from the HCA. Likelihood-ratio chi-square tests were performed for whether cluster explained (1) fruit blush colour, and (2) the presence (n = 127) vs absence of blush (n = 81), irrespective of the intensity of blush. We then removed the individuals with no blush from the dataset to test whether there was a significant difference in fruit blush intensity between clusters for just the individuals with fruit blush using a likelihood-ratio chi-square test with an odds ratio. Finally, we performed a mixed linear model to test the effect of cluster on trunk circumference. JMP v15.2.0 (SAS 2015) produced all statistical results reported here.

## Supplementary Information

Supplementary Information 1.

Supplementary Information 2.

## Data Availability

All data generated or analysed during this study are included in this published article (and its supplementary information files).
