# Supplementary material for: The influence of genetic structure on phenotypic diversity in the Australian mango (Mangifera indica) gene pool
Source: Sci Rep. 2022 Nov 30;12:20614. doi: 10.1038/s41598-022-24800-7 (PMC9712640; doi:10.1038/s41598-022-24800-7)
Supplement: Supplementary file 1 — Supplementary Information 1. [file 41598_2022_24800_MOESM1_ESM.pdf]

## Supplementary Tables and Figures for

### The influence of genetic structure on phenotypic diversity in the Australian mango (M*angifera indica*) gene pool

Melanie J. Wilkinson[*1,2], Risa Yamashita[3], Maddie E. James[1,2], Ian S.E. Bally[4], Natalie L. Dillon[4], Asjad Ali[4], Craig M. Hardner[†3] and Daniel Ortiz-Barrientos[†1,2].

[1]School of Biological Sciences, The University of Queensland, Brisbane, QLD 4072, Australia. [2]Australian Research Council Centre of Excellence for Plant Success in Nature and Agriculture, The University of Queensland, Brisbane, QLD 4072, Australia. [3]Queensland Alliance for Agriculture and Food Innovation, The University of Queensland, Brisbane, QLD 4072, Australia. [4]Queensland Department of Agriculture and Fisheries, Mareeba, QLD 4880, Australia.

Keywords: Evolutionary history, phylogenetic history, horticulture, plant breeding and population structure

[*] Author for correspondence: Melanie J. Wilkinson (m.wilkinson2@uq.edu.au)

† Contributed equally to this work.

**Table S1. Mean and standard error (SE) for trunk circumference for the six geographic regions of import of *M. indica*.**

| Region | N samples | Mean | SE |
|---|---|---|---|
| Africa | 5 | 53.4 | 6.7 |
| Americas | 46 | 52.5 | 1.3 |
| Middle East | 4 | 55.5 | 1.3 |
| Oceania | 54 | 51.1 | 1.1 |
| South Asia | 38 | 48.1 | 1.8 |
| Southeast Asia | 54 | 49.3 | 1.2 |
| Unknown | 7 | 53.1 | 3.9 |

**Table S2. Fruit blush colour percentages for the six geographic regions of import of *M. indica*.**

| Region | no blush | orange | pink | red | burgundy |
|---|---|---|---|---|---|
| Africa | 20 | 0 | 0 | 60 | 20 |
| Americas | 11 | 9 | 13 | 43 | 24 |
| Middle East | 0 | 0 | 25 | 50 | 25 |
| Oceania | 28 | 17 | 17 | 35 | 4 |
| South Asia | 58 | 11 | 16 | 16 | 0 |
| Southeast Asia | 67 | 15 | 15 | 4 | 0 |
| Unknown | 29 | 14 | 29 | 14 | 14 |

**Table S3. Fruit blush intensity percentages for the six geographic regions of import of *M. indica*.**

| Region | no blush | barely visible | slight (Kensington Pride) | medium (Haden) | strong (Tommy Atkins) |
|---|---|---|---|---|---|
| Africa | 20 | 0 | 20 | 40 | 20 |
| Americas | 11 | 2 | 24 | 41 | 22 |
| Middle East | 0 | 50 | 25 | 0 | 25 |
| Oceania | 28 | 22 | 39 | 9 | 2 |
| South Asia | 58 | 24 | 11 | 8 | 0 |
| Southeast Asia | 67 | 28 | 6 | 0 | 0 |
| Unknown | 29 | 0 | 14 | 29 | 29 |

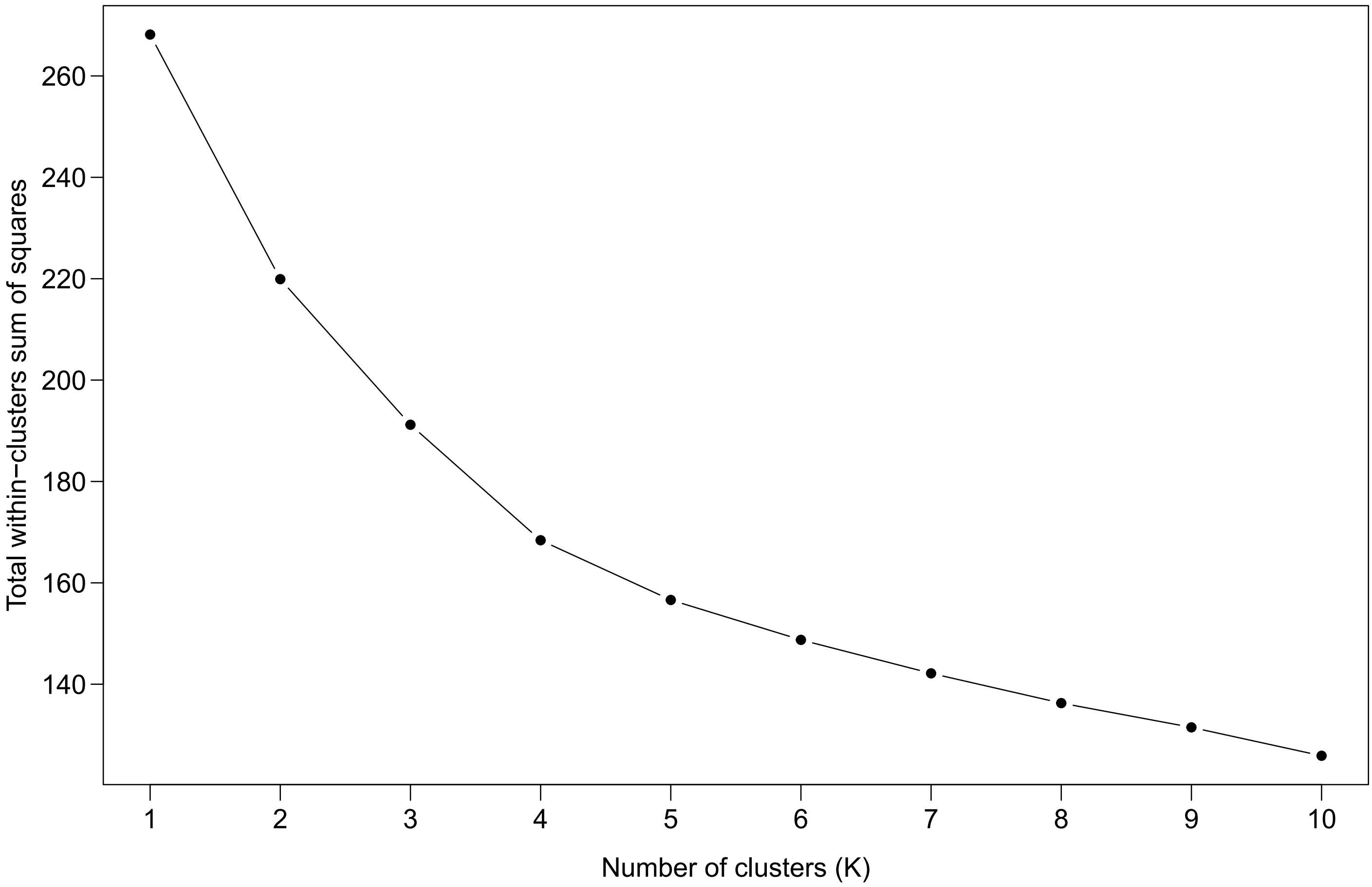


**Fig. S1. The optimal number of clusters (K) using the elbow method.** The point where the rate slows for the total within-clusters sum of squares is the optimal number of clusters derived from the hierarchical cluster analysis.

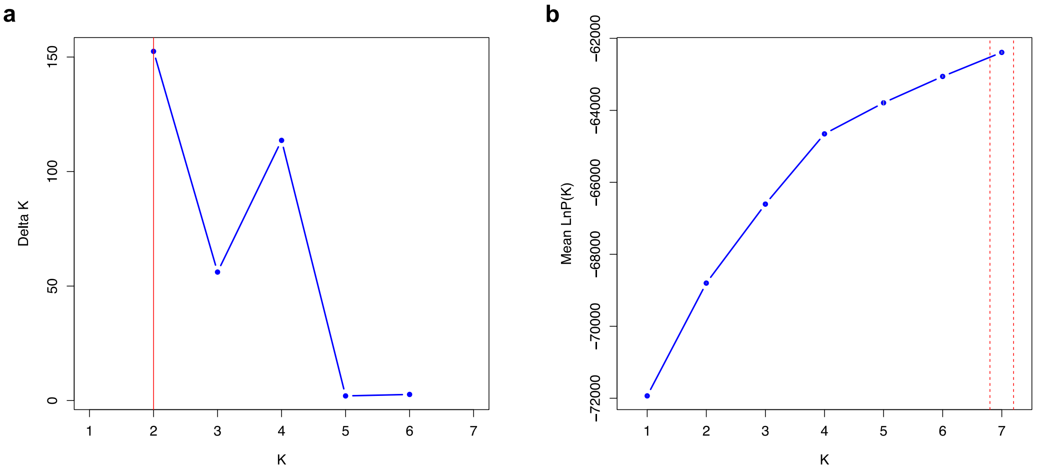


**Fig. S2. STRUCTURE best K values.** STRUCTURE best K values for K=1-7 based on **a)** ΔK (the second order rate of change in the log probability of data between successive K values), and **b)** mean LnP(K) (the mean log probability of the data). Red lines indicate the optimal K values.

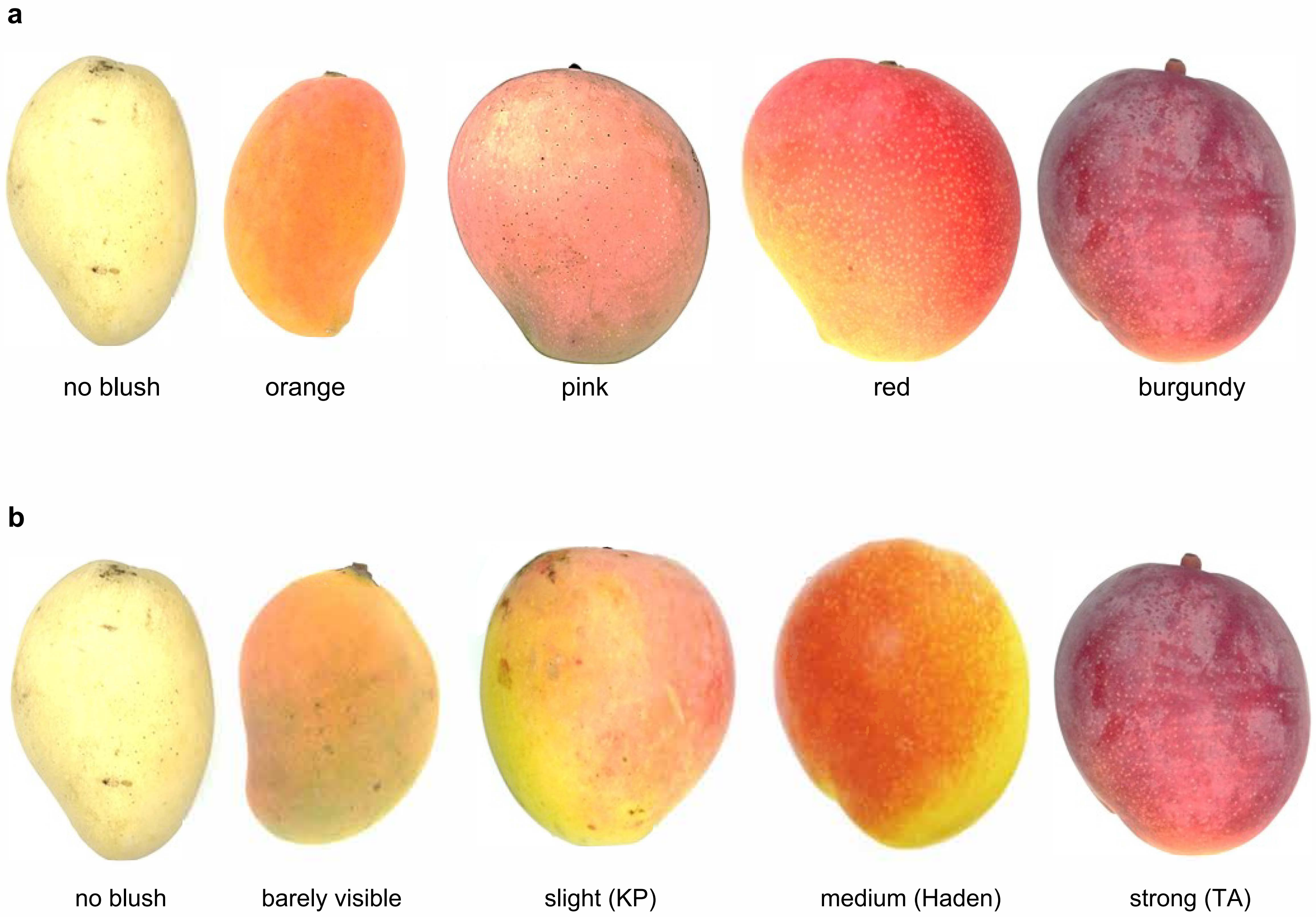


**Fig. S3. Fruit trait categories. a)** Fruit blush colour is split into five categories. **b)** Fruit blush intensity increases from no blush to strong blush on an ordinal scale. The accessions in brackets best reflect the colour intensity, where KP is Kensington Pride and TA is Tommy Atkins.

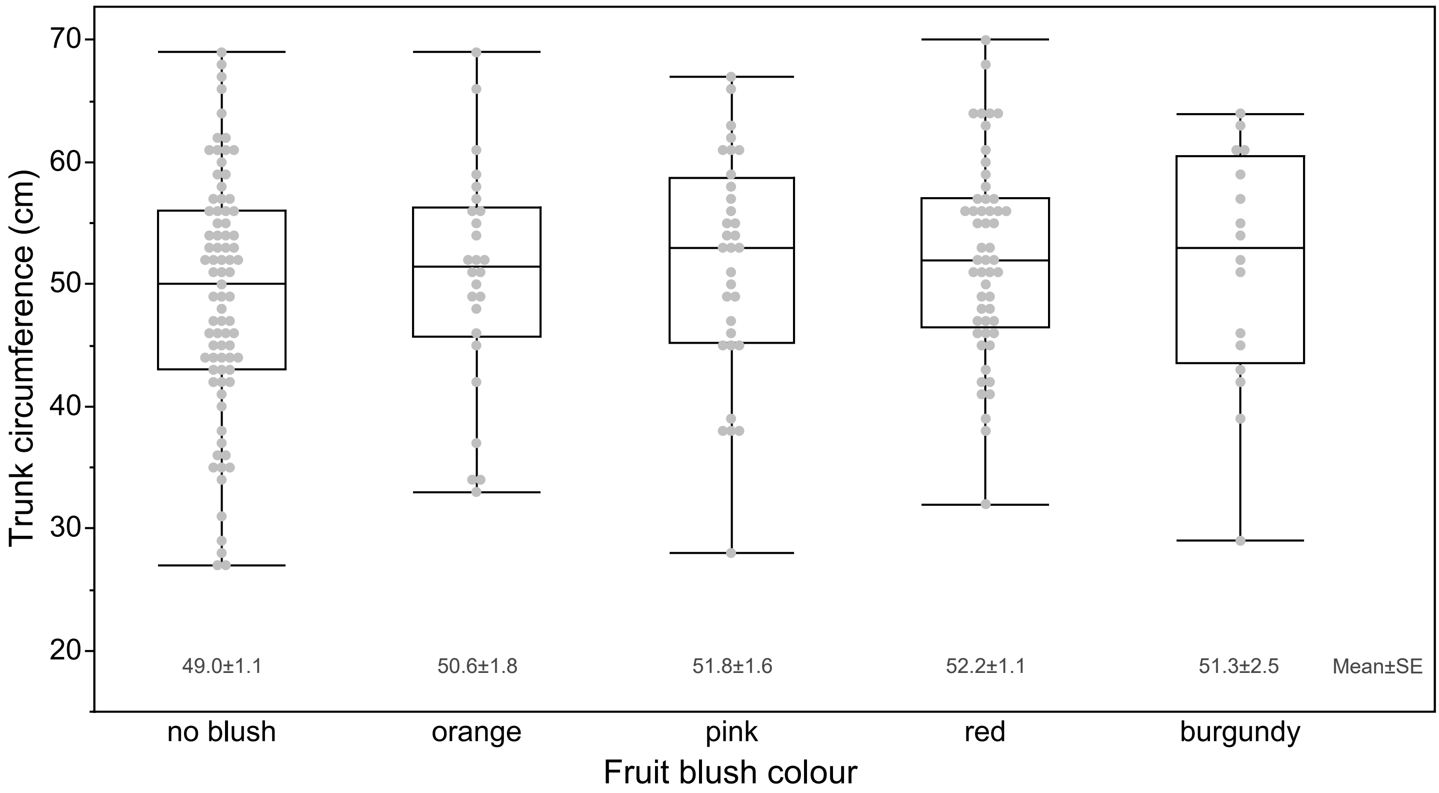


**Fig. S4. The mean and standard error (SE) of trunk circumference across fruit blush colour categories.**

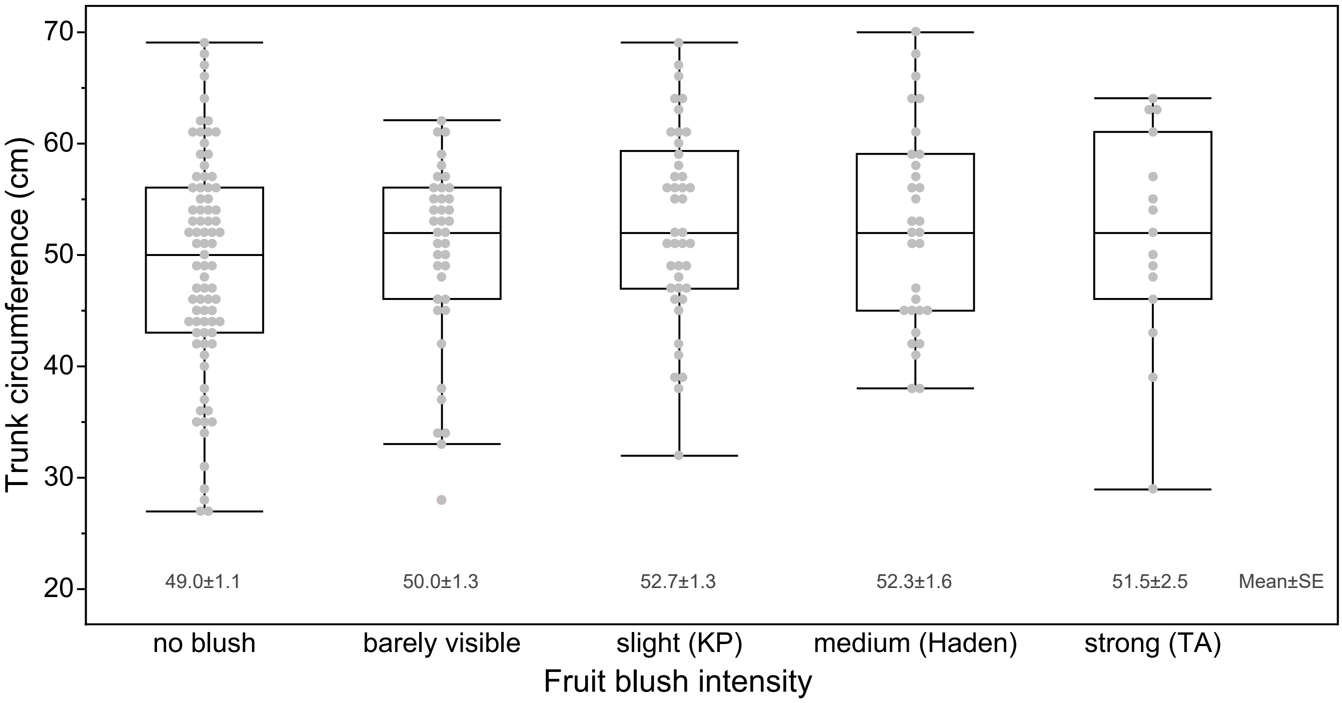


**Fig. S5. The mean and standard error (SE) of trunk circumference across fruit blush intensities.** The accessions in brackets best reflect the colour intensity, where KP is Kensington Pride and TA is Tommy Atkins.
